# Response to luspatercept can be predicted and improves overall survival in the real‐life treatment of LR‐MDS

**DOI:** 10.1002/hem3.70086

**Published:** 2025-02-12

**Authors:** Angela Consagra, Luca Lanino, Najla H. Al Ali, Luis Aguirre, Zhuoer Xie, Onyee Chan, Gloria Andreossi, Marco G. Raddi, Luca Rigodanza, Alessandro Sanna, Giorgio Mattiuz, Elena Tofacchi, Cristina Amato, Michele Tanturli, Sven De Pourcq, Alison Walker, Andrew Kuykendall, Jeffrey Lancet, Eric Padron, David A. Sallman, Francesco Restuccia, Alessandra Perego, Marta Ubezio, Bruno Fattizzo, Marta Riva, Giulia Maggioni, Alessia Campagna, Matteo G. Della Porta, Valeria Santini, Rami S. Komrokji

**Affiliations:** ^1^ MDS Unit, DMSC Hematology University of Florence, AOU Careggi Florence Italy; ^2^ IRCCS Humanitas Research Hospital Milan Italy; ^3^ Department of Biomedical Sciences Humanitas University, Pieve Emanuele Milan Italy; ^4^ Department of Malignant Hematology Moffitt Cancer Center Tampa Florida USA; ^5^ Dana‐Farber Cancer Institute Boston Massachusetts USA; ^6^ Department of Medical Biotechnologies University of Siena Siena Italy; ^7^ Department of Experimental and Clinical Biomedical Sciences “Mario Serio” University of Florence Florence Italy; ^8^ Hematology Unit, Pescara Hospital Pescara Italy; ^9^ Ospedale San Gerardo, ASST Monza Italy; ^10^ Department of Oncology and Onco‐Hematology Fondazione IRCCS Ca' Granda Ospedale Maggiore Policlinico Milan Italy; ^11^ Department of Hematology ASST Grande Ospedale Metropolitano Niguarda Cancer Center Milan Italy

## Abstract

We explored the impact of luspatercept therapy on overall survival (OS) and possible predictors of response in low‐risk (LR) myelodysplastic syndrome (MDS) patients. We evaluated 331 anemic patients treated with luspatercept. Hematological response (HI) was defined as (i) hemoglobin (Hb) increase of ≥1.5 g/dL in nontransfusion‐dependent (NTD) patients, and (ii) red blood cell (RBC) transfusion independence (TI) with a concomitant Hb increase of ≥1.5 g/dL, or RBC‐TI without an Hb increase of 1.5 g/dL, or >50% reduction in RBC transfusion burden (TB) for TD patients. Response was observed in 166 patients (50.2%), with significantly higher response in NTD and low TB versus high TB patients (*p* < 0.001). A significant correlation between lower Molecular International Prognostic Scoring System (IPSS‐M) risk scores and response was observed. No statistically significant difference in HI was found in *SF3B1‐*mutated versus wild‐type MDS patients (53.8% vs. 40.1%, respectively). *SF3B1*mut hotspots (K700E vs. others) and variant allele frequencies (VAFs; <38% VAF vs. ≥38% VAF) did not impact on HI. *SF3B1‐*mutated MDS with del5q showed inferior HI compared to other LR‐MDS (*p* = 0.046). The median treatment duration overall was 35 weeks (20.86–90.29), the median time to response was 11 weeks (8.71–21.86), and the median duration of response was 65 weeks (26.5–114). After a median follow‐up of 13 months, median OS was not reached (NR) for responders and 24 months for nonresponders (hazard ratio [HR] 0.25, 95% confidence interval 0.14–0.44, *p *< 0.001). This analysis of 331 luspatercept real‐life‐treated LR‐MDS patients demonstrated a significant OS benefit upon luspatercept response. Low baseline RBC‐TB and lower risk IPSS‐M scores correlated with higher HI and could constitute predictive markers of response.

## INTRODUCTION

Myelodysplastic syndromes (MDS) or neoplasms are a heterogeneous group of myeloid neoplasms characterized by ineffective hematopoiesis leading to peripheral blood cytopenia and increased risk of progression to acute myeloid leukemia (AML).^1^ MDS is one of the most common causes of anemia in the elderly, and most patients become red blood cell (RBC) transfusion‐dependent (TD) during the course of the disease. Anemia and RBC‐TD exacerbate comorbidities and correlate with shorter survival and reduced quality of life.^2^, ^3^, ^4^, ^5^ Disease management is challenged by heterogeneity in clinical course and variability in long‐term outcomes. Stratifying patient per prognosis is of great importance to optimize therapy. The International Prognostic Scoring System‐Revised (IPSS‐R)^6^ and the novel Molecular International Prognostic Scoring System (IPSS‐M)^7^ are useful for treatment decisions and individual risk assessment. By IPSS‐R,^6^ MDS patients with lower risk (LR‐MDS) are identified by a risk score of <3.5. For these risk categories, treatment aims at improving cytopenias, mainly anemia^8^; however, more recently, the concept of the relevance of prolonged survival has gained attention.

Erythropoiesis‐stimulating agents (ESAs) are used to treat symptomatic anemia in LR‐MDS with reported erythroid responses in 30%–40% of cases.^9^ Luspatercept, trapping TGF‐β superfamily Activin B ligands and enhancing late‐stage erythropoiesis, improves anemia, and it was approved by the Food and Drug Administration (FDA) and the European Medicines Agency (EMA) in RBC‐TD LR‐MDS‐RS post‐ESA failure based on the results of Medalist study, where transfusion independence (TI) for 8 weeks or longer was observed in 38% of patients receiving luspatercept,^10^ and subsequently it was compared to ESA as the first line for all subtypes of LR‐MDS RBC‐TD in the COMMANDS trial.^11^ First results of real‐world use of luspatercept were recently published,^12^, ^13^, ^14^ and low transfusion burden (LTB) was confirmed to correlate with higher response to the agent.

We explored the impact of luspatercept on overall survival (OS). We also investigated possible factors correlated with response to luspatercept in the largest cohort of treated LR‐MDS patients in hematological centers adhering to Fondazione Italiana Sindromi Mielodisplastiche (FISiM) and in Moffitt Cancer Center (MCC).

## METHODS

### Patients

Patients with a diagnosis of LR‐MDS (with RS in the majority of cases, as well as some RS negative, according to 2016 WHO criteria^15^) and treated with luspatercept were retrospectively collected. Patients from the MCC cohort were treated since FDA approval, while those from FISiM were included in the multicenter observational trial (registered at ClinicalTrials.gov, NCT05520749)^10^ as a compassionate use program. A small number of patients (*n *= 7) with MDS non‐RS were also included (MCC cases) (Table 1). Baseline blood cell counts, morphologic, and molecular bone marrow data were defined as the closest ones determined prior to the first dose of the drug. Anemic MDS patients were classified as either TD or nontransfusion‐dependent (NTD) based on their RBC transfusion requirements in the 8 weeks prior to treatment initiation, interval indicated in IWG 2006.^14^ We modified the definition of TD to indicate patients who in our cohort received any RBC transfusion within 8‐week period. To further specify baseline transfusion burden (RBC‐TB), we categorized patients as follows: NTD (0 units in 8 weeks prior to luspatercept), LTB (1–5 units in 8 weeks), and high transfusion burden (HTB; ≥6 units in 8 weeks).

**Table 1:** Characteristics of patients.

	MCC‐FISiM (*n* = 331)
Age (median)	75 (31–94)
Gender (male)	211 (63.74)
Hb (mean), g/dL	7.97 (5.5–11.5)
PLT (mean), ×10^9^/L	268.5 (15–1002)
ANC (mean), ×10^9^/L	2.86 (0.38–13.5)
Serum erythropoietin level (median), U/L	60.1 (*n* = 111)

WHO 2016 (*n* = 331)	*n* (%)
MDS‐RS	310 (96.5)
MDS‐del5q	5 (1.5)
MDS‐MLD	2 (0.6)
MDS/MPN with RS and thrombocytosis	14 (4.4)

NGS	*n* (%)
*SF3B1*	169/181 (93.4)
*U2AF1*	6/171 (3.5)
*ZRSR2*	7/171 (4.1)
*TET‐2*	57/171 (33.3)
*DNMT3A*	38/171 (22.2)
*ASXL‐1*	25/171 (14.6)
*TP53*	11/171 (6.4)
*EZH‐2*	8/171 (4.7)
*ETV‐6*	3/171 (1.7)
*SETBP1*	10/171 (5.8)
*RUNX‐1*	6/171 (3.5)
*CBL*	2/171 (1.1)
*JAK‐2*	16/171 (9.3)

IPSS‐M (*n* = 154)	*n* (%)
Very low	1 (0.0064)
Low	93 (60.38)
Moderate low	33 (21.42)
Moderate high	20 (12.98)
High	6 (3.89)
Very high	1 (0.0064)

IPSS‐R (*n* = 291)	*n* (%)
Very low	10 (3.43)
Low	239 (82.13)
lntermediate	37 (12.71)
High	5 (1.71)
Very high	‐

RBC transfusion burden	*n* (%)
NTD	21 (6.3)
LTB	126 (38.1)
HTB	184 (55.6)

Prior treatment	*n* (%)
ESA	317/331 (95.77)
HMA	52/331 (15.7)
Lenalidomide	38/331 (11.5)

Line of treatment with luspatercept	*n* (%)
First	9/331 (2.7)
Second	263/331 (79.5)
Third	32/331 (9.7)
Fourth	27/331 (8.1)

*Note*: Demographic and clinical features are reported; diagnosis according to the WHO 2016 classification, somatic mutations, and prognostic score according to the revised IPSS and molecular IPSS.

Abbreviations: ANC, absolute neutrophil count; ESA, erythropoiesis‐stimulating agents; Hb, hemoglobin; HMA, hypomethylating agents; HTB, high transfusion burden (≥6 units/8 weeks); IPSS‐M, Molecular International Prognostic Scoring System; IPSS‐R, Revised International Prognostic Scoring System; LTB, low transfusion burden (1–5 units/8 weeks); MDS‐del5q, myelodysplastic syndrome with deletion of 5q; MDS‐MLD, myelodysplastic syndrome with multilineage dysplasia; MDS/MPN with RS and thrombocytosis, myelodysplastic syndrome/myeloproliferative neoplasm with ring sideroblasts and thrombocytosis; MDS‐RS, myelodysplastic syndrome with ring sideroblasts; NTD, nontransfusion dependent (0 units in 8 weeks prior luspatercept; PLT, platelets.

### Treatment

Luspatercept was administered according to package insert instructions^10^ at the recommended starting dose of 1 up to 1.75 mg/kg, once every 3 weeks by subcutaneous injection. Patients who had received at least one dose of luspatercept were included. Patients were followed for a median follow‐up of 13 months (9–16 months). The follow‐up was updated on June 30, 2023. Survival analysis included censoring at the time of AML evolution or hematopoietic stem cell transplant (HSCT).

### Response criteria

Hematologic improvement‐erythroid (HI‐E) in NTD patients was defined as an increase in hemoglobin (Hb) levels of at least 1.5 g/dL, sustained for a minimum of 8 weeks. We defined HI‐E response in TD patients dividing into two categories: (1) RBC‐TI with a Hb increase of ≥1.5 g/dL, and (2) RBC‐TI without a Hb increase of 1.5 g/dL, both sustained for a minimum of 8 weeks. We also report the reduction of >50% in RBC‐TB among RBC‐TD patients.

## STATISTICAL ANALYSIS

Clinical and genetic data LR‐MDS patients were analyzed using R software version 4.3.3 (Angel Food Cake) with R Studio RStudio 2023.12.1 + 402 “Ocean Storm”; a *p* value of <0.05 was considered statistically significant. The associations between categorical variables were calculated by Fisher's exact test, and when applicable using the Chi‐square test; odds ratio (OR) with a confidence interval at 95% level (95% CI) was also reported. In cases where continuous variables were assessed, a nonparametric approach was employed by means of the Wilcoxon/Mann–Whitney test and the Kruskal–Wallis test, reporting median and interquartile range (IQR). Survival analysis was performed with the Kaplan–Meier method together with the log‐rank test and Cox proportional hazards regression. The latter was also used for hazard ratios (HR) with 95% CI calculation and multivariate analyses for adjusting for relevant covariates. Generalized Linear Models were also used for multivariate analyses. The choice of relevant covariates, used for adjusted multivariate analyses, was based on clinical and biological relevance together with the Akaike information criterion (AIC). Finally, in the case of multiple comparisons, Holm correction was used.

## RESULTS

### Baseline characteristics

Between March 2016 and March 2023, 331 LR‐MDS patients were included in the study. Baseline demographic and clinical characteristics are presented in Table 1. Overall, the median age at luspatercept treatment inception was 75 years; 120/331 patients (36%) were female. Median percentages of BM myeloblasts and RS were 1% (1%–2%) and 30% (16%–55%), respectively. The determination of age‐adjusted cellularity pre‐treatment by bone marrow trephine was known in 179/331 patients: 74.3% of cases were hypercellular, 3.4% hypocellular, and 22.3% normocellular.

Karyotype, presence of somatic mutations by NGS analysis, and evaluation of IPSS‐R^6^ and IPSS‐M^7^ were obtained. *SF3B1* mutation was present in 169/181 (93.4%). Hotspot mutations were known in 142/169 patients, and K700E was the most common one observed (53.5%, 76/142); median variant allele frequency (VAF) was 38.35% (2.6–53.05). A more complete NGS mutational panel was evaluated in 171 patients, and the most common co‐mutated genes were *TET2* (33.3%), *DNMT3A* (22.2%), and *ASXL1* (14.6%). *TP53* was mutated in 6.4% of patients with a VAF ranging from 5% to 49%. According to the 2016 WHO criteria,^15^ 310/331 patients (94%) had MDS with RS, while 151/181 (83%) met the criteria for diagnosis of MDS with mutation of *SF3B1*, according to the 2022 WHO criteria.^16^ In this cohort, there were also five patients with the diagnosis of MDS with del5q, who harbored *SF3B1* mutation, and 14 patients with MDS/MPN with RS and thrombocytosis. As highlighted in Table 1, 239/291 (82.13%) and 93/154 (60.38%) of patients had a low risk in accordance with IPSS‐R^6^ and IPSS‐M,^7^ respectively. Performance status prior to treatment initiation was assessed using the Eastern Cooperative Oncology Group (ECOG) scale and was known for 188 out of 331 patients, which is distributed as follows: ECOG 0 for 39.4% of patients, ECOG 1 for 53.7%, and ECOG 2 for 6.9%. Twenty‐one (6.3%) patients were RBC‐NTD before the treatment, while 126/331 (38.1%) and 184/331 (55.6%) had LTB and HTB, respectively. The baseline median TB was 6 units/8 weeks (0–22). The median line of treatment with luspatercept was in the second line (with a range between the first and fourth lines). Prior therapy included ESAs (95.8%), lenalidomide (11.5%), and HMAs (15.7%). Overall, 27/331 patients (8.1%) received luspatercept as the fourth line of therapy. Dose escalation to 1.33 and 1.75 mg/kg occurred in 80.5% and 73.5% of patients, respectively.

### Response

Erythroid response according to the abovementioned criteria observed in 166 patients (50.2%) (Supporting Information S1: Table 1) is as follows: 23.9% of patients obtained a Hb increase of ≥1.5 g/dL in the NTD group or a similar increase with RBC‐TI within RBC‐TD category; 16.6% of patients achieved an RBC‐TI without experiencing Hb increase of >1.5 g/dL, while 9.7% exhibited a reduction of >50% in TB.

Erythroid response, as previously defined, was significantly higher in NTD and LTB patients (*p* < 0.001) compared to HTB patients. Specifically, 81% (17/21) of NTD patients achieved HI‐E, while among those with LTB, 32.5% achieved RBC‐TI with increase in Hb of ≥1.5 g/dL. RBC‐TI was achieved in 17.5% of cases, and 7.1% achieved a 50% reduction in transfusion requirement. Regarding the HTB group, the overall response was observed in 41.8% of patients (77/184), which is distributed as follows: 11.4% obtained an increase in Hb of 1.5 g/dL plus RBC‐TI, 17.9% achieved RBC‐TI, and 12.5% had >50% reduction in transfusion requirement.

The patients who received luspatercept as the second line after ESA were 259, and their ORR was 51.3% (133/259): 45.1% (60/133) of patients obtained TI with Hb increase ≥1.5 g/dL, 36.8% (49/133) achieved RBC‐TI without experiencing Hb increase >1.5 g/dL, 18% (24/133) exhibited a reduction >50% in TB. Luspatercept response was relevant even when used as the third line: 16/38 (42%) of patients pretreated with ESAs and lenalidomide (*p* = 0.078) and 23/52 (44.2%) of those pretreated with ESAs and HMA (*p* = 0.047) responded. Efficacy was also observed in fourth‐line therapy (patients pretreated with ESAs, lenalidomide, and HMA): 40.7% (11/27) of cases, *p* = 0.141.

The median duration of treatment in the entire population was 35 weeks (20.86–90.29), while in responders it was 80.5 weeks (44.25–125.04). The median time to response was 11 weeks (8.71–21.86), while the median duration of response was 65 weeks (26.5–114).

At the last follow‐up, 57.7% of patients (191/331) had discontinued treatment: 126 patients discontinued treatment due to lack of response, 26 due to loss of response, 6 due to adverse events, 10 due to disease progression, and 23 due to death. Regarding patients treated within the FISiM protocol, only data on major adverse events were collected: 35 patients (17.4%) presented serious adverse events (SAE), not drug related. The most frequent SAEs included cardiac events (such as hypertension, acute heart failure, and atrial fibrillation; *n* = 11), acute kidney injury (*n* = 1), infections (*n* = 10), COVID‐19 pneumonia (*n* = 4), and falls resulting in bone fractures (*n* = 4). At the last follow‐up, a total of six patients had proceeded to HSCT.

### Predictive factors

For 154/331 patients with calculated IPSS‐M prior to luspatercept, the response was significantly correlated with disease risk, as shown in Table 2 (*p* = 0.031), while the IPSS‐R score did not correlate with the response (*p* = 0.247). Among 179 cases with available BM biopsy, 74.3% were hypercellular, 3.4% were hypocellular, and 22.3% were normocellular (age‐adjusted cellularity). Response was obtained in 76/133 patients (57.1%) with hypercellular marrow and 16/46 (34.8%) with normocellular or hypocellular.

**Table 2:** Response to treatment and relationship with prognostic score and *SF3B1* mutational status.

	Response	No response	*p* Value
IPSS‐M, *n* (%)			
Very low	1 (1.2)	0 (0.0)	0.031
Low	56 (70.0)	37 (50.0)	
Moderate low	12 (15.0)	21 (28.4)	
Moderate high	10 (12.5)	10 (13.5)	
High	1 (1.2)	5 (6.8)	
Very high	0 (0.0)	1 (1.4)	
IPSS‐R, *n* (%)			
Very low	6 (4.2)	4 (2.7)	0.247
Low	123 (85.4)	116 (78.9)	
Intermediate	13 (9.0)	24 (16.3)	
High	2 (1.4)	3 (2.0)	
*SF3B1* status, *n* (%)			
*SF381*mut	91 (91.0)	78 (85.7)	0.267
*SF381* WT	9 (9.0)	13 (14.3)	
*SF3B1* hotspot, *n* (%)			
K700E	45 (57.7)	31 (48.4)	0.312
Others	33 (42.3)	33 (51.6)	
*SF3B1* VAF, *n* (%)			
≥38%	37 (52.1)	42 (66.7)	0.113
<38%	34 (47.9)	21 (33.3)	
*SF3B1* co‐mutations, *n* (%)			
*SF3B1* ^*α*^	72 (90.0)	57 (82.6)	0.046
*SF3B1* ^*β*^	8 (10.0)	7 (10.1)	
*SF3B1* ^*5q*^	0 (0.0)	5 (7.2)	

*Note*: Response to luspatercept according to IPSS‐M, IPSS‐R, SF3B1 mutational status, SF3B1 hotspot, SF3B1 median VAF of our cohort, and SF3B1 co‐mutations groups according to the 2022 edition of WHO classification and IPSS‐M.

Abbreviations: IPSS‐M, Molecular International Prognostic Scoring System; IPSS‐R, Revised International Prognostic Scoring System; *SF3B1^α^*, as any other mutant; *SF3B1* ^*β*^, *SF3B1* and any gene from *BCOR, BCORL1, NRAS, RUNX1, SRSF2*, or *STAG2*; *SF3B1* ^*5q*^, concomitant presence with isolated del5q; VAF, variant allele frequency.

In relation to BM cellularity, patients with hypercellular bone marrow had a trend to respond better to treatment compared to the hypocellular and normocellular groups (*p* = 0.005, OR 1.5, CI 0.27–8.48). Pre‐treatment ECOG performance status did not influence drug response (*p *= 0.611).

We also evaluated the response (both TI or ORR) in relation to the presence of the *SF3B1* mutation (Table 2). There was no statistically significant difference in response rate (*p* = 0.267, OR 1.69, CI 0.68–4.15) in *SF3B1*‐mutated patients compared to wild‐type ones, 91/169 (53.8%) versus 9/22(40.1%), respectively. We did not observe a statistically significant difference when we evaluated response in relation to *SF3B1* mutation hotspots (K700E vs. others) (*p* = 0.312, OR 0.69, CI 0.35–1.34), nor when median VAF was evaluated by stratifying <38% VAF versus ≥38% VAF (*p* = 0.113, OR 1.84, CI 0.91–3.70). Segregation of *SF3B1*‐mutant cases into three distinct groups, according to the IPSS‐M and WHO 2022, revealed a varying HI rate. *SF3B1* ^*β*^ (*SF3B1* and any gene from *BCOR, BCORL1, NRAS, RUNX1, SRSF2*, or *STAG2*) and *SF3B1* ^*α*^ (*SF3B1* mutation alone or with any other mutant different from the previous group) demonstrated superior erythroid improvement rates compared to *SF3B1* ^*5q*^ (concomitant presence of mutation and isolated del5q): 8/15 (53.8%), 72/129 (55.8%), and 0/5 (0%) respectively, with a significant *p* = 0.046. In the MDS non‐RS subgroup, one patient with a diagnosis of MDS‐MLD achieved TI, although this was not accompanied by an increase in Hb levels exceeding 1.5 g/dL. Another patient with MDS‐MLD showed no response.

**Figure 1:**
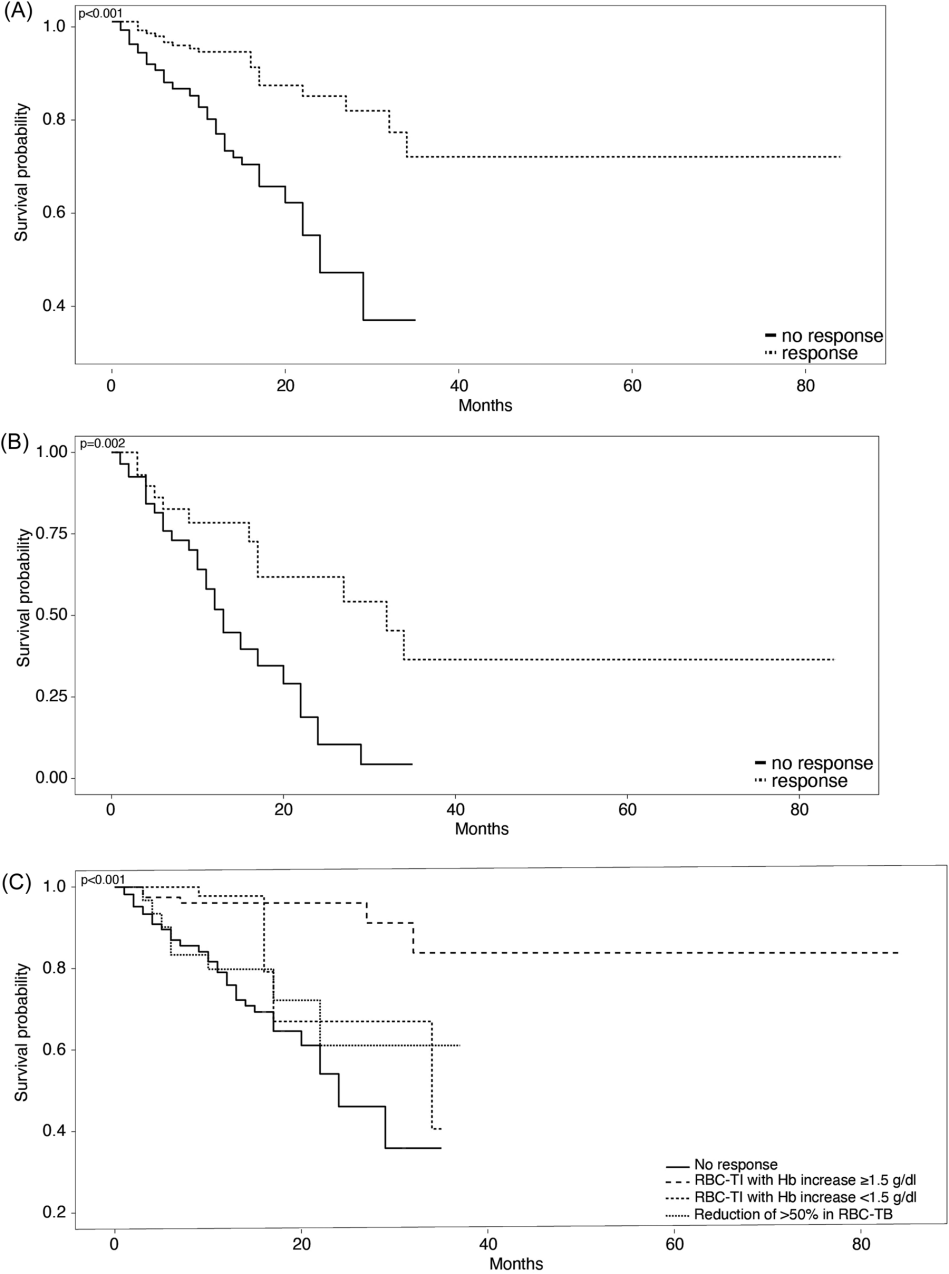
**Kaplan–Meier estimates of survival by response**. With a median follow‐up of 13 months from starting luspatercept treatment, the median overall survival (OS) for all treated patients was not reached (NR) (95% confidence interval [CI] 0.32 to NA). **(A)** OS by response to luspatercept (hazard ratio [HR] 0.25, 95% CI 0.14–0.44, *p *< 0.001); **(B)** OS by response to luspatercept adjusted for red blood cell (RBC) transfusion burden (<6 RBC/8 weeks or ≥6 RBC/8 weeks), IPSS‐M and IPSS score (HR 0.30, 95% CI 0.14–0.64, *p* = 0.002); **(C)** OS by type of response to luspatercept (solid line: no response; dashed line: RBC‐TI with hemoglobin (Hb) increase of ≥1.5 g/dL; dashed/dotted line: RBC‐TI without Hb increase of ≥1.5 g/dL; dotted line: reduction of more than 50% in RBC transfusion burden among RBC transfusion‐dependent patients) (HR 0.12, 95% CI 0.05–0.31, *p* < 0.001). RBC‐TI, red blood cell transfusion independence.

Multiple logistic regression analysis was conducted to explore the relationship between the likelihood of attaining a primary response and the baseline characteristics of the patients. The multivariate analysis revealed that both IPSS‐M and the burden of RBC transfusions had the most predictive value (Supporting Information S1: Table 2). IPSS‐M was then maintained as a continuous variable, while the optimal categorical separation for predicting RBC transfusion was identified as 0, <6, or ≥6 units of packed RBC per 8 weeks (Figure 1B).

### OS

With a median follow‐up of 13 months from starting luspatercept treatment, the median OS for all treated patients was NR (95% CI 0.32 to NA).

The median OS was NR among responders to luspatercept compared to 24 months for nonresponders (HR 0.25, 95% CI 0.14–0.44, *p* < 0.001) (Figure 1A). In multivariable analysis adjusted for IPSS‐M, IPSS‐R, and baseline RBC‐TB (<6 RBC/8 weeks or ≥6 RBC/8 weeks), response to luspatercept remained associated with improved OS (HR 0.30, 95% CI 0.14–0.64, *p *= 0.002) (Figure 1B).

The median OS benefit was more pronounced among patients who obtained RBC‐TI and Hb ≥ 1.5 g/dL increase compared to other categories of responders and nonresponders. A statistically significant difference in OS was observed in patients with RBC‐TI and Hb ≥ 1.5 g/dL versus patients with no response or reduction in TB (HR 0.12, 95% CI 0.05–0.31, *p *< 0.001) (Figure 1C and Supporting Information S1: Table 3).

**Figure 2:**
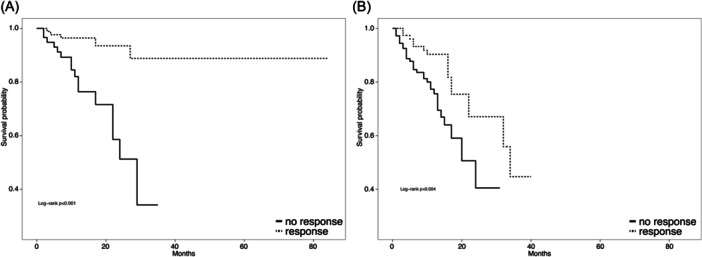
**Kaplan–Meier estimates of survival by response dividing patients for transfusion burden. (A)** Overall survival in NTD/LTB patients by luspatercept response (hazard ratio [HR] 0.15, 95% confidence interval [CI] 0.05–0.40, *p* < 0.001); **(B)** overall survival in HTB patients by luspatercept response (HR 0.37, 95% CI 0.19–0.74, *p* = 0.005). HTB, high transfusion burden (≥6 units/8 weeks); LTB, low transfusion burden (1–5 units/8 weeks); NTD, nontransfusion dependent (0 units in 8 weeks prior luspatercept).

Based on baseline RBC‐TB, the median OS was NR among NTD/LTB responders compared to 29 months among nonresponders (HR 0.15, 95% CI 0.05–0.40, *p *< 0.001) (Figure 2A and Supporting Information S1: Table 4). In the HTB group, the median OS for luspatercept responders was 34 months compared to 24 months for nonresponders (HR 0.37, 95% CI 0.19–0.74, *p *= 0.005) (Figure 2B and Supporting Information S1: Table 5).

## DISCUSSION

Luspatercept has been approved by FDA and EMA for treating anemia in adult patients diagnosed with LR‐MDS, who require RBC transfusions (≥2 units every 8 weeks).^17^, ^18^ The first approval was limited to patients with MDS‐RS after ESA failure, while more recently luspatercept was approved for all LR‐MDS subtypes also in first‐line treatment.^10^, ^19^ We present here the outcome of the largest real‐world data set of LR‐MDS patients receiving luspatercept in the second line of therapy and beyond (up to the fourth line of therapy). Our study in fact included LR‐MDS patients who received previous therapies beyond ESAs, such as lenalidomide and/or HMAs. Treatment was also administered regardless of patient frailty. TI >8 weeks was observed in 117/310 cases (37.7%), consistent with the results of the registration trial. Real‐world studies are fundamental to confirm the benefit of the agents evaluated beyond the clinical trial given that eligibility criteria for prospective phase II and phase III trials are typically very stringent, not representing patients receiving those treatments in clinical practice.

We confirm the activity of luspatercept in RW as previously published in controlled studies.^6^ In the present study, a small number of LR‐MDS non‐RS cases, as well as MDS with del5q with *SF3B1*mut, have been included providing important data needed to address the response of MDS non‐RS to luspatercept post‐ESA failure as the COMMANDS study did not demonstrate the superiority of luspatercept to ESA in the frontline setting, raising the important question of the responses based on the sequence of therapy of luspatercept/ESA versus ESA/luspatercept.

We demonstrated that RBC TB at baseline is the most important predictor of response. This observation stresses the importance of early treatment of symptomatic anemia. A randomized phase III clinical trial (registered at ClinicalTrials.gov, NCT05949684) is ongoing to evaluate the efficacy of luspatercept versus ESAs in transfusion‐independent LR‐MDS patients.

When considering the mutational pattern of LR‐MDS cases, low scoring according to IPSS‐M correlated with response. In the LR‐MDS cohort analyzed here, neither the presence of specific *SF3B1* hot spot mutation nor its VAF correlated with response. Stratification of patients based on co‐mutation subgroups, as delineated by IPSS‐M, demonstrated an improved response trend in the *SF3B1* ^*α*^ group, suggesting that the co‐mutation pattern may influence response to treatment, besides having prognostic significance. Patients in the *SF3B1* ^del5q^ subgroup exhibited poor response to luspatercept and to our knowledge this is the first report in this sense, and it has special interest prompting further investigations. While the small sample size limits the statistical power of these findings, it is noteworthy, given that in the luspatercept clinical trials (MEDALIST^10^ and COMMANDS^11^), patients with MDS with del5q were not included. Our preliminary observation is the first in this setting, and it seems in fact to discourage the use of luspatercept based merely on the presence of *SF3B1* mutation in MDS with del5q patients. In fact, the functional consequences of such splicing mutation could differ profoundly as it is mirrored by the negative prognostic value of *SF3B1* mutation in MDS^del5q^ (as well as in other diseases such as CLL). More data are awaited as a clinical study is ongoing for MDS with del5q and *SF3B1* mutation (registered at ClinicalTrials.gov, NCT05924100).^20^

The evaluation of OS in LR‐MDS undergoing specific treatments has only recently become a subject of interest.^21^, ^22^, ^23^ RBC‐TD is associated with worse OS, reflecting disease biology and complications of prolonged RBC transfusions.^2^ Achievement of RBC TI had been suggested to correlate with better survival, independently from the type of therapy (i.e., ESAs, lenalidomide, and luspatercept); the median OS was significantly higher in responders versus no responders.^21^, ^22^, ^23^ We acknowledge that assessing OS based on response can be perceived as a bias, but we reckon that this observation has a strong clinical significance. In the present study, median OS was NR among responders to luspatercept, compared to 24 months in nonresponders (*p *< 0.001). We also demonstrated that the quality of response can be associated with better outcomes. Patients achieving TI and experiencing Hb increase of ≥1.5 g/dL had prolonged OS compared to patients who achieved only RBC‐TI or significant reduction in transfusion need.

The present analysis, encompassing a larger cohort of LR‐MDS patients treated with luspatercept, represents the largest study conducted to date mirroring the real‐world experience of more than 300 patients. Since this was a retrospective study, patient responses were checked and controlled by a manual review of medical records. Data were maintained prospectively only for the cases still followed up in our centers, which may have introduced some limitations in accurately capturing the totality of responses. As a result, there is a possibility that some responses could be either overestimated or missed. Although limitations of this study include its retrospective nature, as well as the lack of complete somatic mutation data, we believe that it provides validation in heterogenous LR‐MDS population of what is observed in clinical trials, and indicates possible avenues of investigation in specific subgroups of MDS.

## AUTHOR CONTRIBUTIONS

Angela Consagra, Luca Lanino, Valeria Santini, and Rami S. Komrokji designed the study, analyzed data, and wrote the manuscript. Michele Tanturli performed statistical analysis. Giorgio Mattiuz participated in the analysis and optimized tables and figures. All the authors collected data and approved the final version of this manuscript.

## CONFLICT OF INTEREST STATEMENT

The authors declare the following competing financial interests: V. S. has participated in advisory boards for Abbvie, Ascentage, BMS, Geron, Jazz, Keros, Novartis, Servier, and Syros, and has received travel grants from Abbvie and Jazz. R. K. has received consulting fees from Geron; has received research funding from Bristol Myers Squibb; has participated in advisory boards for AbbVie, Bristol Myers Squibb, CTI Biopharma, Jazz Pharmaceuticals, Novartis, PharmaEssentia, Rigel, Servio, and Taiho; and has received payment for speakers bureaus from AbbVie, CTI Biopharma, Jazz Pharmaceuticals, PharmaEssentia, and Servio. O. C. has worked for BMS and has received research funding from AbbVie and Jazz. M. G. D. P. has participated in advisory boards for BMS. E. P. has participated in advisory boards for BMS, GSK, Stemline, SoBi, Pharmaessentia, and Taiho and has received research funding from BMS and Incyte. D. A. S. has participated in consulting or advisory roles for Celyad, Agios, Abbvie, Aprea AB, Bristol‐Myers Squibb, Gilead Sciences, Intellia Therapeutics, Kite Pharma, Magenta Therapeutics, Novartis, and Syndax Speakers' Bureau for Agios, Incyte, and Bristol‐Myers Squibb; and has received research funding from Celgene and Jazz Pharmaceuticals. A. K. has participated as a consultant for MorphoSys, Abbvie, Karyopharm, Protagonist, and GSK and has received Honoraria from Incyte, Silence Therapeutics, Imago Biosciences, GSK, and BMS, as well as research support from Novartis, Geron, Janssen, GSK, BMS, Protagonist, and Blueprint.

## FUNDING

This study was partially supported by the Associazione Italiana per la Ricerca sul Cancro (AIRC) Investigator Research Grant (IG‐26 537‐2021) to V. S.

## Supporting information

Supporting information.

## Data Availability

The data sets generated and analyzed during the current study are available from the corresponding author upon reasonable request.
