# Supplementary material for: Response to luspatercept can be predicted and improves overall survival in the real‐life treatment of LR‐MDS
Source: Hemasphere. 2025 Feb 12;9(2):e70086. doi: 10.1002/hem3.70086 (PMC11814532; doi:10.1002/hem3.70086)

**Supplementary**

**Table-S1: different type of responses according to transfusion burden**


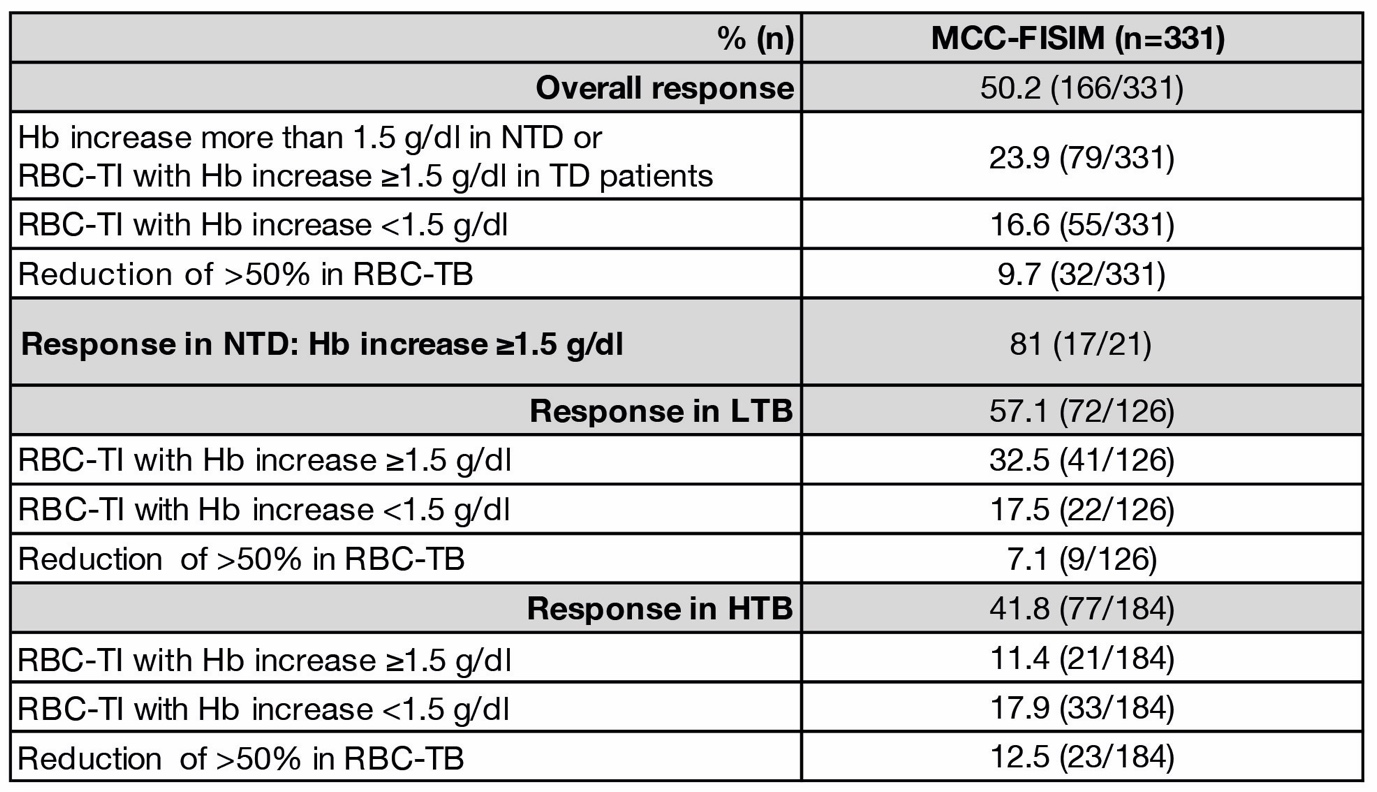


Abbreviations: RBC-TI, red blood cell – transfusion independence; Hb, hemoglobin; NTD, non-transfusion dependent (0 units in 8 weeks prior luspatercept); LTB , low transfusion burden (1-5 units/8 weeks); HTB, high transfusion burde (≥ 6 units/8 weeks).

**Table-S2: Response Vs no-response: a multivariates model**


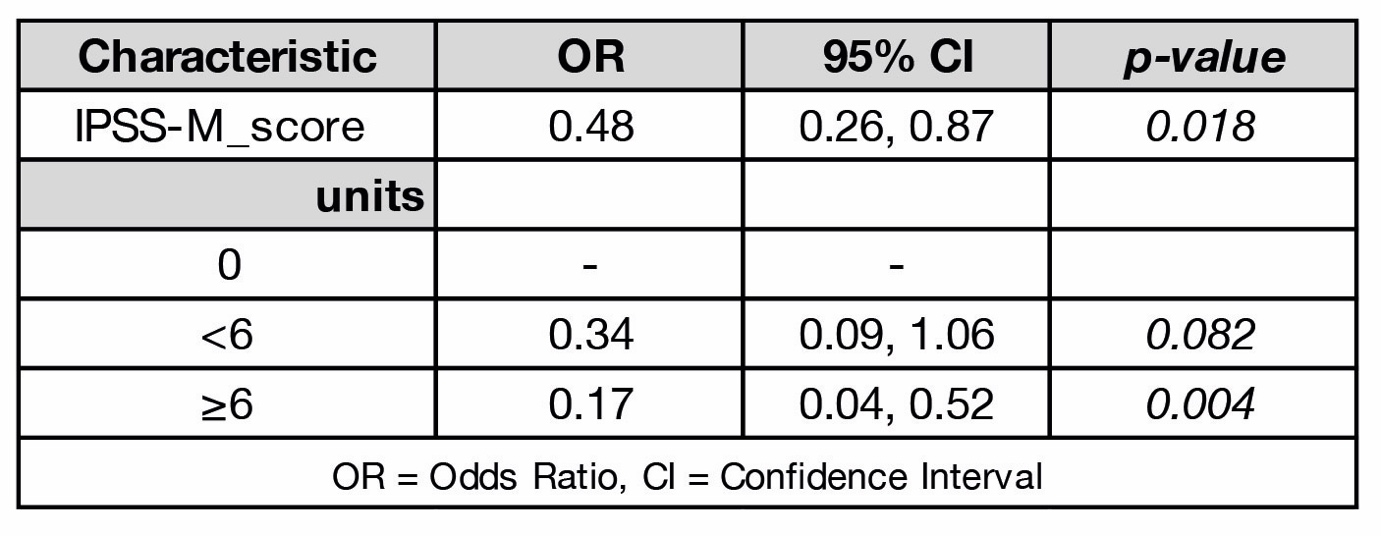


Abbreviations: IPSS-M, Molecular International Prognostic Scoring System.

**Table-S3: HR of figure 1C**

**
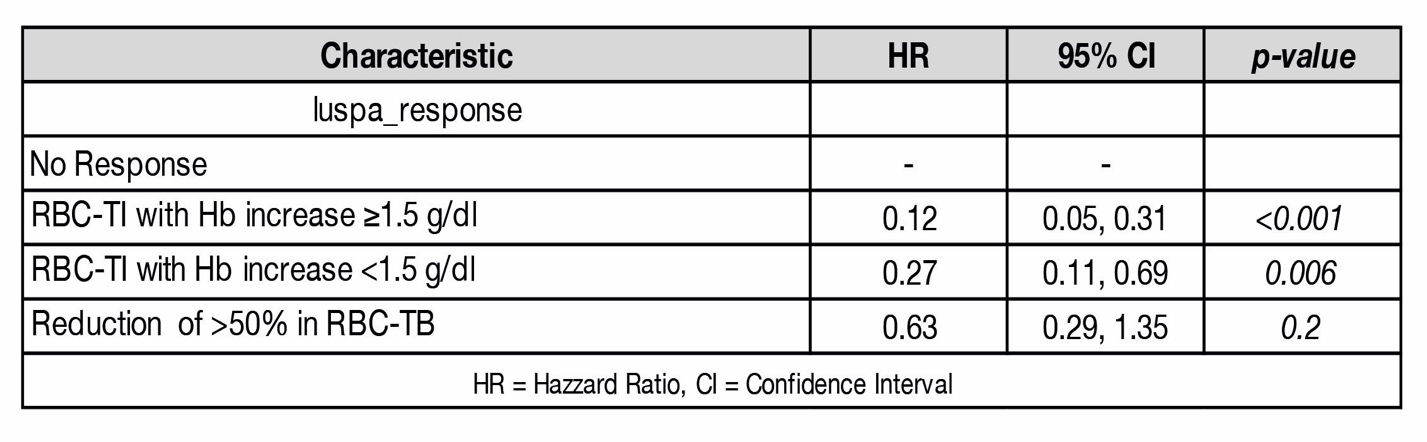
**

Abbreviations: RBC-TI, red blood cell – transfusion independence; Hb, hemoglobin

**Table-S4: HR of figure 2A**

**
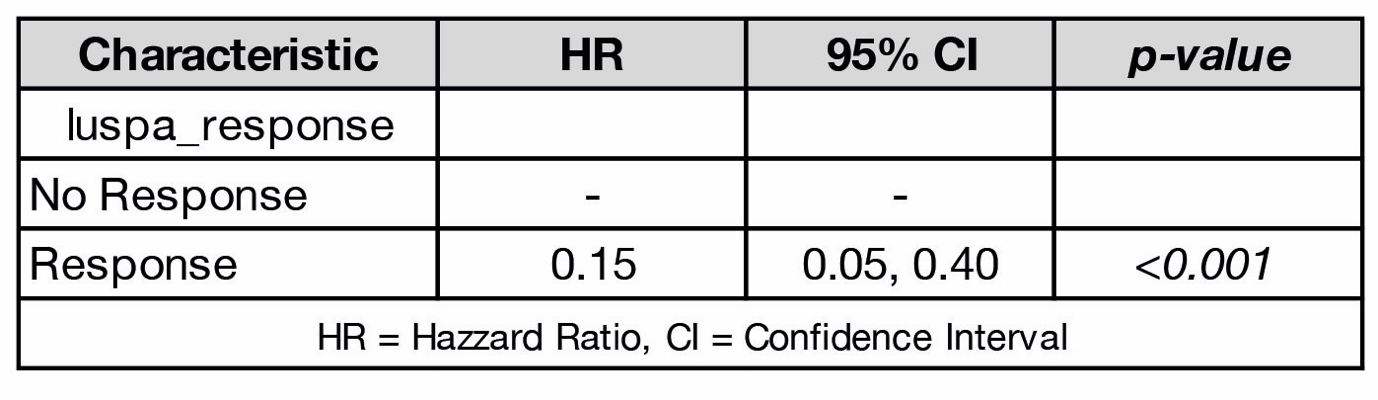
**

**Table-S5: HR of figure 2B**


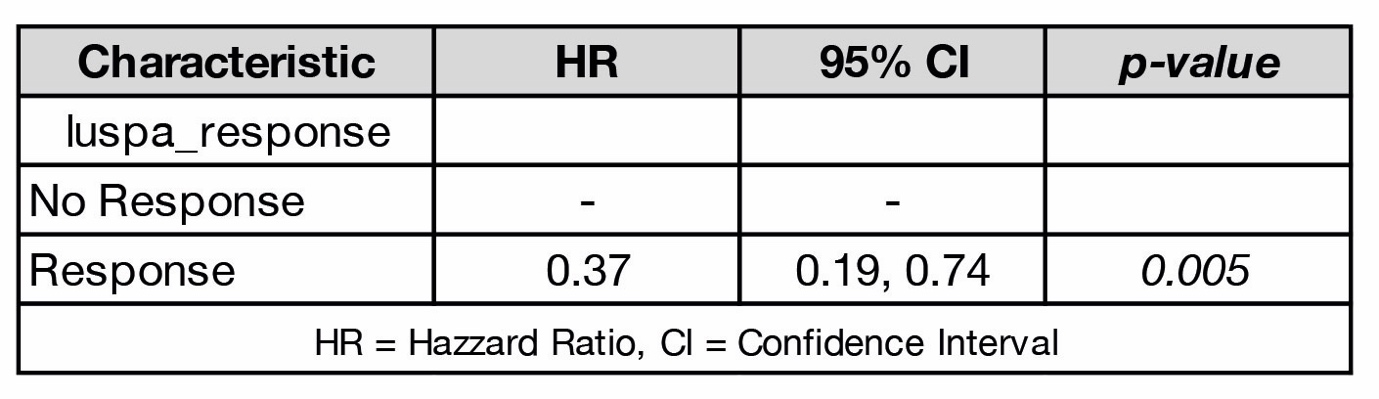

Supplement: Supplementary file 1 — Supporting information. [file HEM3-9-e70086-s001.docx]
